# Treatment of *Klebsiella Pneumoniae* Carbapenemase (KPC) infections: a review of published case series and case reports

**DOI:** 10.1186/1476-0711-11-32

**Published:** 2012-12-13

**Authors:** Grace C Lee, David S Burgess

**Affiliations:** 1 Pharmacotherapy Education & Research Center, School of Medicine, University of Texas Health Science Center at San Antonio, 7703 Floyd Curl Drive – MC 6220, San Antonio, TX 78229-3900, USA; 2 College of Pharmacy, University of Texas at Austin, 1 University Station, Austin, TX 78712, USA; 3 Department of Pharmacy Practice and Science, University of Kentucky College of Pharmacy, 789 S. Limestone, 292K, Lexington, KY, 40536, USA

**Keywords:** KPC, Treatment outcome, *Klebsiella pneumoniae* carbapenemases, Carbapenemase, Polymyxin, Carbapenems, Tigecycline, Aminoglycosides

## Abstract

The emergence of *Klebsiella pneumoniae* carbapenemases (KPCs) producing bacteria has become a significant global public health challenge while the optimal treatment remains undefined. We performed a systematic review of published studies and reports of treatment outcomes of KPC infections using MEDLINE (2001–2011). Articles or cases were excluded if one of the following was fulfilled: no individual patient data provided, no treatment regimen specified, no treatment outcome specified, report of colonization, or greater than three antibiotics were used to treat the KPC infection. Data extracted included patient demographics, site of infection, organism, KPC subtype, antimicrobial therapy directed at KPC-infection, and treatment outcome. Statistical analysis was performed in an exploratory manner. A total of 38 articles comprising 105 cases were included in the analysis. The majority of infections were due to *K. pneumoniae* (89%). The most common site of infection was blood (52%), followed by respiratory (30%), and urine (10%). Forty-nine (47%) cases received monotherapy and 56 (53%) cases received combination therapy directed at the KPC-infection. Significantly more treatment failures were seen in cases that received monotherapy compared to cases who received combination therapy (49% vs 25%; p= 0.01). Respiratory infections were associated with higher rates of treatment failure with monotherapy compared to combination therapy (67% vs 29% p*=* 0.03). Polymyxin monotherapy was associated with higher treatment failure rates compared to polymyxin-based combination therapy (73% vs 29%; p*=* 0.02*)*; similarly, higher treatment failure rates were seen with carbapenem monotherapy compared to carbapenem-based combination therapy (60% vs 26%; p*=* 0.03). Overall treatment failure rates were not significantly different in the three most common antibiotic-class combinations: polymyxin plus carbapenem, polymyxin plus tigecycline, polymyxin plus aminoglycoside (30%, 29%, and 25% respectively; p=0.6). In conclusion, combination therapy is recommended for the treatment of KPC infections; however, which combination of antimicrobial agents needs to be established in future prospective clinical trials.

## Introduction

The increasing incidence of *Klebsiella pneumoniae* carbapenemases (KPCs) is a significant public health challenge [1]. Previously confined to sporadic outbreaks in the northeastern United States, KPCs have now spread throughout the world and have reached endemic proportions in countries such as Israel and Greece [2,3]. Furthermore, KPC-producing organisms can confer resistance to multiple different antimicrobial classes, including all available β-lactams, fluoroquinolones, and aminoglycosides [4,5]. As such, infections due to KPCs are associated with high therapeutic failure and mortality rates of at least 50% [6,7]. The limited number of agents available for the treatment of KPCs presents a tremendous challenge to clinicians. Given the lean pipeline of new antimicrobials, further investigations into optimal treatment modalities are urgently needed. However, studies on the treatment of KPC infections are scarce and mainly limited to case series and case reports. Therefore, we sought to perform a systematic review of individual cases in an effort to summarize therapeutic outcomes of various treatment regimens for KPC infections.

### Case selection

A systematic review of English language articles using MEDLINE (2001–2011) was conducted. Additional studies were identified by searching bibliographies of primary articles and annual conference abstracts from 2008–2011. Search terms included kpc.mp, Drug Therapy/mt, mo, Treatment Outcome, Case Reports, and Disease Outbreaks/pc. All searches were limited to humans. Articles were eligible if they included patients with infections due to KPC-producing bacteria. Articles were excluded from further review if they fulfilled at least one of the following criteria: no individual patient data reported, no treatment regimen specified, no treatment outcome specified, or greater than three antibiotics or multiple antibiotic regimens directed at the KPC infection. Clinical success and failures were recorded as reported by the authors of each report. Analysis of the proportion of clinical failures was calculated as the number of failures divided by the number of treated patients. Several characteristics from the cases were extracted including the patient’s age, sex, medical history, site(s) of infection, type of infection, organism, KPC subtype, APACHE II score, admission to the intensive care unit (ICU), length of stay before infection, total length of stay, minimum inhibitory concentration (MIC) of selected antimicrobials (carbapenem, polymyxin, and tigecycline), antimicrobial therapy before isolation, antimicrobial therapy directed at KPC-infection, and treatment outcome. Antimicrobial agents were categorized into the following classes: polymyxins, carbapenems, glycylcycline, aminoglycosides, cephalosporins, beta-lactam plus beta-lactamase inhibitors, fluoroquinolones, trimethoprim-sulfamethoxazole, monobactams, fosfomycin, and tetracyclines. Combination therapy was defined as at least two but no more than three, antibiotics with gram-negative activity reported to be directed at KPC infections. Statistical analysis was performed in an exploratory manner. Comparisons were made using χ^2^ or Fisher's exact test for categorical variables using JMP 8.0 ® (SAS Corp, Cary, NC).

#### Study characteristics

**Figure 1:**
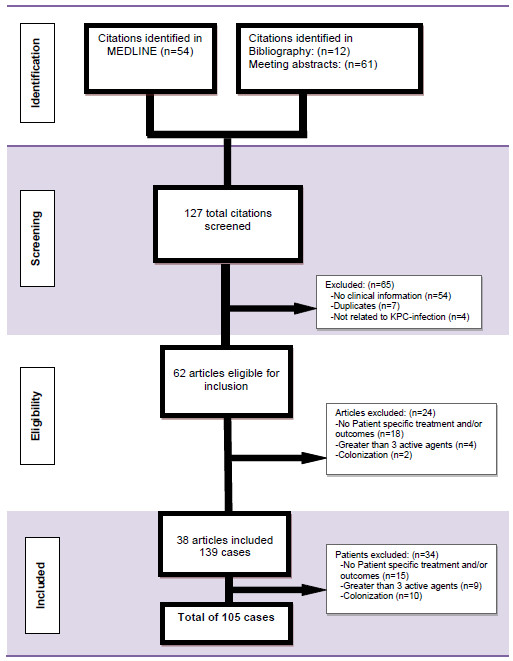
Case Selection.

A total of 54 relevant articles were identified searching MEDLINE, 12 from bibliographies of retrieved articles, and 61 from conference abstracts (Figure 1). Of these 127 citations, 62 articles were eligible for review for study inclusion. A total of 24 articles were excluded for the following reasons: no patient specific treatment and/or outcomes (n=18), greater than three antimicrobials (n=4), and reports of colonization (n=2). Within the 38 articles included, 34 individual cases were excluded for the following reasons: no patient specific treatment and/or outcomes (n=15), greater than three antimicrobials (n=9), and reports of colonization (n=10). A total of 38 reports comprising 105 cases met the inclusion criteria, and consisted of case series (47%), retrospective cohort studies (35%), and case reports (17%). The majority of reports were from the US (42%), followed by Greece (10%). As expected, the number of studies reported per year increased. The majority of the articles (58%) for this review were published in 2009 and 2011.

Several limitations should be noted. Individual studies/reports did not always define treatment failure or treatment success. Antimicrobial dosing, procedures, residual colonization, antimicrobial timing were not always reported and therefore not considered in the outcome analysis.

#### Characteristics of cases

Characteristics of cases evaluated in this review are presented in Table 1. The mean age was 62 years ± 19, 55% were males, and 72% were admitted to the ICU. The mean APACHE II Score was 20.6 ± 8, the mean length of stay before infection was 17.5 days ± 19, while the mean total length of stay was 54.8 days ± 40. Infection characteristics are described in Table 2. Most of the reported infections were due to KPC-2 (86%). Organisms largely comprised of *K. pneumoniae* (89%), followed distantly by *Pseudomonas spp* (4%), *E. coli* (3%), *S. marcescens* (3%), and *E. cloacae* (2%). The most common site of infection was blood (52%), followed by lung (30%), and urine (10%).

**Table 1:** Patient characteristics

**Characteristic**	
Age n = 88^a^	62 ±19
Male Gender n(%) n = 60^a^	33 (55)
ICU admission n(%) n = 57^a^	41(72)
APACHE II Score n = 38^a^	20.6±8
LOS before infection n = 48^a^	17.5±19
LOS, total n = 24^a^	54.8±40

Abbreviations: *ICU*: Intensive care unit; *APACHE II*: Acute Physiology and Chronic Health Evaluation II; *LOS*: length of stay.

Data reported as mean ± standard deviation unless otherwise noted.

^a^Number of study subjects for which this data were provided.

**Table 2:** Infection characteristics

**Characteristic**	**Data available (n)**	**Number of cases (%)**
KPC-subtype	98	
KPC-2		84(86)
KPC-3		14(14)
Organisms^a^	104	
*K. pneumoniae*		92(89)
*E. coli*		3(3)
*S. marcescens*		3(3)
*Pseudomonas spp*		4(4)
*E. cloacae*		2(2)
Site of infection	105	
Blood		56(53)
Pulmonary		32(31)
Urine		11(11)
Skin/Wound		4(4)
Cerebral spinal fluid		1(1)
Bone		1(1)

^a^ Specific organism was not reported in 1 case.

^b^ Column and row percentages may not total 100 due to rounding error.

#### Treatment of KPC infections

Cases received regimens containing the following antimicrobials or antimicrobial classes: 45 cases received a polymyxin, 39 cases received a carbapenem, 30 cases received an aminoglycoside, 26 cases received a glycylcycline, 7 cases received a fluoroquinolone, 5 cases received a beta-lactam plus beta-lactamase inhibitor, 2 cases received cephalosporins, 2 cases received a tetracycline, 2 cases received a monobactam, 1 case received fosfomycin, and 1 case received trimethoprim-sulfamethoxazole. Overall, 49 (47%) cases received monotherapy and 56 (53%) cases received combination therapy directed at KPC-infections.

#### Summary of treatment outcomes

A summary of the treatment outcomes is provided in Table 3. The overall rate of treatment failure was 36% (38/105). Pulmonary infections were associated with the highest rate of treatment failure (47%) followed by blood infections (39%). Of the cases with KPC infections due to *Klebsiella species*, 62% (57/92) reported treatment success, 50% (1/2) for *Enterobacter cloacae*, 100% (3/3) for *Serratia marcescens,* and 100% (3/3) for *E. coli*. Interestingly, all 4 cases of *Pseudomonal* KPC infections reported treatment failure. Infections with *bla*_kpc-3_ had a higher rate of treatment failure than *bla*kpc-2 (36% vs. 50%; p = 0.35). Pathogens harboring *bla*_kpc-3_ consisted of *K. pneumoniae* (12; 86%) and *Raoultella spp* (2; 14%)*.*

**Table 3:** Overall treatment outcome

	**Data available**	**Treatment outcome (%)**
Overall treatment failure	105	38/105 (36)
Source (failure)	105	
Blood		22/56(39)
Pulmonary		15/32(47)
Urine		1/11(9)
KPC-type	98	
KPC-2		30/84(36)
KPC-3		7/14(50)

#### Polymyxins

Polymyxin monotherapy had higher rates of treatment failure compared to polymyxin-based combination therapy (73% vs 29%; p*=* 0.02*)*[8-24]. Of the 11 cases who received polymyxin monotherapy, 73% (8/11) experienced treatment failure [8,10,18,20,23,25]. Of the nine patients treated for bloodstream infections (BSIs), six patients were treatment failures. Both patients treated for pneumonia with colistin monotherapy failed treatment [18]. Among cases who received polymyxin-based combination therapy, 71% (24/34) patients had successful treatment outcomes. Of these 24 patients, the most common treatment regimen was tigecycline plus colistin (50%), followed by carbapenem plus polymyxin (33%) and aminoglycosides plus polymyxin (13%). Twenty-nine percent of patients (10/34) who received polymyxin-based combination therapy experienced treatment failure. Five patients were treated for BSIs with the following regimens: carbapenem plus colistin (3), tigecycline plus colistin (1), and amikacin plus colistin (1) [8,9,12]. In the latter case, the patient developed colistin resistance (MIC 0.25 μg/mL to 16 μg/mL) while on treatment and eventually died. The other five cases of treatment failure consisted of cases of pulmonary infection treated with the following antimicrobials in combination with a polymyxin: tigecycline (3), carbapenem (1), and fluoroquinolone (1) [8,11,16,26].

Although polymyxins were the most commonly used antimicrobial against KPC infections in this review, the effectiveness of polymyxins for the treatment of KPC infections is not well established. Various in-vitro studies have demonstrated bactericidal activity and synergy when polymyxins are tested in combination with carbapenems, rifampin or tigecycline, while monotherapy often led to significant re-growth by 24 hours [27-29]. Furthermore, a report by Lee and colleagues [30] evaluated 12 patients with persistent blood cultures positive for KPC-*K. pneumoniae* despite 3 days of treatment with either polymyxin B alone or polymyxin B plus tigecycline. Interestingly, 3 of the 12 patients who received polymyxin B monotherapy had significant increases in MIC values (1.5 μg/mL to 32 μg/mL, 0.75 μg/mL to 12 μg/mL, and 0.75 μg/mL to 1024 μg/mL) while none of the patients who received polymyxin B in combination with tigecycline developed resistance. The authors hypothesized that combination therapy may have prevented resistance in patients who received both polymyxin B and tigecycline. Similar reports of gram negative organisms developing resistance to colistin have been described [31-33]. Polymyxins are one of the few antimicrobial classes that remain susceptible to KPC-producing organisms. Increasing reports of polymyxin resistance described in case reports and recent outbreaks of polymyxin-resistant strains are troubling [33-39]. Combination therapy may be an important strategy for the management of KPC infections when utilizing polymyxins. However, determining which antimicrobial in combination with polymyxins is superior still needs to be established in further clinical studies.

#### Carbapenems

Of the 39 cases who received a carbapenem, 51% (20/39) received carbapenem monotherapy and 49% (19/39) received carbapenem-based combination therapy [8,14,16,17,40-50]. Carbapenem monotherapy had higher rates of treatment failure compared to carbapenem-based combination therapy (60% versus 26% p=0.03*)*. Of the 20 cases who received carbapenem monotherapy, 55% (11/20) failed treatment, including 6 cases with strains having MICs ≤4 μg/mL (previous CLSI breakpoint for imipenem and meropenem). The majority of those who failed treatment had pneumonia or tracheobronchitis (7), followed by BSI (3), and urinary tract infection (UTI) (1) [40,43,48,49]. Among cases who received carbapenem-based combination therapy, 74% of patients (14/19) had successful treatment outcomes. In these 14 patients, the most common treatment regimen was polymyxin plus carbapenem (8), followed by aminoglycoside plus carbapenem (4), and beta-lactam plus beta-lactamase inhibitor plus carbapenem (2). A total of 5 patients receiving carbapenem-based combination therapy experienced treatment failure. All but one of these 5 patients were treated with a carbapenem plus polymyxin.

Several issues regarding the use of carbapenems for the treatment of KPC-infections should be considered. Nine of the cases who received carbapenem monotherapy were reported from one retrospective case series [40]. In the retrospective review, patients infected with imipenem non-susceptible KPC *K. pneumoniae* (MIC > 4 μg/mL) treated with alternative therapies had a higher rate of successful treatment (8/10, 80%) when compared with patients infected with imipenem-susceptible (MIC ≤ 4 μg/mL) KPC *K. pneumoniae* treated with a carbapenem (4/9, 44.4%). The optimal pharmacodynamic parameter for carbapenems is the time the free carbapenem concentration remains above the MIC (%*T* > MIC) [51]; however, whether this parameter is valid in the presence of KPCs is controversial. In a mouse thigh infection model, Craig *et al.*[52] demonstrated that the presence of KPCs had no impact on achieving pharmacodynamic parameters of strains with MICs up to 16 μg/mL. There has been only one case report describing the successful use of high-dose continuous infusion meropenem monotherapy for a carbapenem non-susceptible KPC *K. pneumoniae* (MIC= 8 μg/mL) BSI [50]. In contrast, an *in vitro* model, simulating human exposure to a high-dose prolonged-infusion meropenem (2 g every 8 h over 3-hr infusion), demonstrated that the killing activity at 24 hours, against KPC-producing *K. pneumoniae* strains, were inferior to non-carbapenemase-producing *Pseudomonas aeruginosa* strains despite similar meropenem MICs [53]. The authors attributed this to the rapid in-vitro hydrolysis of meropenem, resulting in lower exposure of meropenem to the KPC *K. pneumoniae*. The Clinical and Laboratory Standards Institute (CLSI) recently lowered clinical breakpoints of carbapenems to exclude KPC-producing organisms in the previous susceptible range [54]. Susceptibilities to imipenem, meropenem and doripenem have been defined by an MIC up to 1 μg/mL and 0.5 μg/mL for ertapenem. The utility of carbapenem monotherapy against strains containing KPCs remains controversial. Until further data are available, the use of combination or alternative therapy may be warranted.

#### Tigecycline

Of the 26 cases who received tigecycline, 7 cases received tigecycline monotherapy and 19 received tigecycline-based combination therapy [8,9,11,12,17,26,40,41,47,55,56]. There was no significant difference in treatment failure rates between cases receiving tigecycline monotherapy compared to those receiving combination therapy (29% vs. 37%;p *=* 0.4*)*. Twenty-nine percent (2/7) of patients who received tigecycline monotherapy experienced treatment failure. One patient was treated for urosepsis and the other was treated for nosocomial pneumonia and empyema [40,56]. In the latter, even though a long course of tigecycline resolved the pneumonia, treatment was complicated by recurrence of empyema associated with an increase in tigecycline MIC from 0.75 to 2 μg/mL. Of the 19 patients who received tigecycline combination therapy, 14 (74%) received polymyxin plus tigecycline, 3 (16%) received tigecycline plus aminoglycoside, and 2 (11%) received tigecycline plus carbapenem. Both patients who received tigecycline plus a carbapenem experienced treatment failures [40,41]. In one report, despite a month-long course of tigecycline and meropenem for KPC-*K. pneumoniae* bacteremia secondary to sclerosing cholangitis, the patient experienced relapse of the bacteremia which was associated with the development of resistance to both meropenem and tigecycline [41].

Tigecycline has demonstrated excellent spectrum of activity against KPC-producing organisms. In a collection of 106 carbapenemase-producing strains from various countries, tigecycline was the only antimicrobial with 100% activity [5]. However, there are no breakpoints set by the CLSI for tigecycline for Enterobacteriaceae (FDA-approved breakpoint for tigecycline is < 2 μg/mL). In addition, tigecycline has limited activity against *Pseudomonas* species. Clinical issues with tigecycline’s pharmacokinetic properties may raise some caution in treatment of UTIs and BSIs due to low urinary (< 22%) and low plasma (≤ 0.9 mg/L) concentrations [57,58]. However, tigecycline has been used successfully in treating these infections; both patients included in this review who were treated with tigecycline monotherapy for their UTIs, were treated successfully [40,59]. In contrast, in an evaluation of 48 patients with carbapenem-resistant *K. pneumoniae* bacteremia in a tertiary care hospital, all 8 patients with break-through bacteremias had received tigecycline [60] reports of tigecycline resistance have been previously demonstrated in *Enterobacteriaceae*[56,61-63]. Finally, tigecycline is a bacteriostatic agent; thus, alternative agents or combination therapy may be required for infections requiring bactericidal activity.

#### Aminoglycosides

A total of 30 cases received an aminoglycoside, 20% (6/30) of cases as monotherapy and 80% (24/30) as combination therapy [8-13,16,19,22,40,42,47,48,55,64-68]. There was no significant difference in treatment failure rates between those who received aminoglycoside monotherapy compared to combination therapy (0% vs. 17%; p*=* 0.6). Interestingly, all six cases who received aminoglycoside monotherapy reported success: three cases were treated for BSIs, two cases were treated for UTIs, and one case was treated for a respiratory infection [11,13,40,64]. A recent study demonstrated that aminoglycosides, when active in vitro, were associated with a significantly higher rate of microbiologic clearance of carbapenem-resistant *K. pneumoniae* in the urine compared to polymyxin B or tigecycline [69]. Of the patients who received aminoglycoside-based combination therapy, the most common treatment was amikacin plus colistin (11), followed by aminoglycoside plus a carbapenem (4), aminoglycoside plus a fluoroquinolone (5), aminoglycoside plus tigecycline (2), gentamicin plus aztreonam (1), and amikacin plus tetracycline (1). Despite in-vitro evidence of antagonism when using polymyxins and aminoglycosides in combination, 6 clinical cases of polymyxin plus an aminoglycoside reported treatment success [11,19,22,65]. All patients who failed therapy with aminoglycoside-based therapy had BSIs. One reported case had bacteremia due to KPC-2-producing *E. cloacae* and *P. putida* recovered simultaneously from multiple cultures; the patient eventually died [68]. Herein, the most commonly employed aminoglycoside combination was amikacin plus colistin; but, the potential increased risk of nephrotoxicity with this combination is a major concern.

#### Combination versus monotherapy

Of all cases meeting the study inclusion criteria, 49 (47%) cases received monotherapy and 56 (53%) cases received combination therapy directed at KPC-infections. Significantly more treatment failures were seen in cases treated with monotherapy than those treated with combination therapy (49% vs. 25%; p= 0.01) (Table 4). Similar treatment failure rates were observed in the three most common antibiotic-class combinations: polymyxin plus a carbapenem, polymyxin plus tigecycline, polymyxin plus an aminoglycoside (30%, 29%, and 25% respectively; p=0.6). In three cases, triple antimicrobial therapy directed at the KPC infection was used: one case received doripenem plus polymyxin B plus rifampin and 2 cases received tigecycline plus colistin plus garamycin [11,17]. All three cases were treated successfully.

**Table 4:** Treatment failure: Monotherapy vs. Combination therapy

	**Monotherapy (%)**	**Combination (%)**	***P***
Overall treatment failure	24/49(49)	14/56(25)	0.01
Source:			
Blood	12/24 (50)	9/32(28)	0.09
Pulmonary	10/15(67)	5/17(29)	0.03
Urine	1/8(13)	0/3(0)	0.4
Polymyxin treatment failure	8/11(73)	10/34(29)	0.02
Carbapenem treatment failure	12/20(60)	5/19(26)	0.03
Tigecycline treatment failure	2/7(29)	7/19(37)	0.4
Aminoglycoside treatment failure	0/6(0)	4/24(17)	0.6

A review of treatment outcomes versus sites of infection showed urinary sources of infection had the highest overall treatment success rates of 81% (9/11). A recent retrospective cohort study of 21 patients with UTIs described similar positive clinical responses in 16 (76%) patients [70]. Of the 11 patients who were treated for UTIs in this review, the majority were treated with monotherapy (8/11), and of these patients, only one experienced treatment failure while on imipenem (MIC=4 μg/mL) [40]. The eight cases treated successfully with monotherapy received either a carbapenem (3), tigecycline (2), an aminoglycoside (1), ciprofloxacin (1) or cefepime (1) [8,16]. All 3 cases treated with combination therapy for UTIs reported treatment success. The pulmonary site of infection was associated with higher rates of treatment failure with monotherapy compared to combination therapy (67% vs. 29% p*=* 0.03). Among the 17 patients with pulmonary infections treated successfully, more than 75% of cases received combination therapy. The most common regimen for the successful treatment of pulmonary infections consisted of tigecycline plus colistin (8), followed by an aminoglycoside plus colistin (3). Likewise, there was a lower failure rate treating blood-stream infections with combination therapy compared to monotherapy, (28% vs. 50%; p=0.09).

Several recent studies have supported the role of combination therapy for treating KPC infections. In a cohort of 41 patients with KPC- *K. pneumoniae* bacteremia, the use of combination therapy was associated with improved 28-day mortality [71]. The most common successful combination employed in this cohort was a polymyxin in combination with either tigecycline or a carbapenem. Tumbarello et al. [72] conducted a study in three Italian hospitals and demonstrated that combination therapy, particularly a triple-drug regimen including tigecycline, colistin, plus a carbapenem, was independently associated with improved survival. Likewise, in a previous pool of 55 individual cases, combination therapy was associated with successful outcomes compared to monotherapy, particularly if polymyxins were part of the regimen [73].

## Conclusion

Infections caused by KPC-producing bacteria have been associated with high mortality rates and frequent treatment failure. Clinical data on treatment are limited and appropriate therapy for KPC infections is not well defined. This review demonstrated that monotherapy is associated with higher treatment failure rates compared to combination therapy when managing infections due to KPC-producing bacteria, particularly when treating respiratory infections. Polymyxins and carbapenems when used alone were associated with higher treatment failure than when used in combination. Until further data are available, combination therapy is recommended. However, which antimicrobial combination is superior has yet to be established.

## Competing interests

The authors declare that they have no competing interests.

## Authors’ contributions

GL carried out the systematic review, performed the analysis, and drafted the manuscript. DB participated in the design and helped to draft the manuscript. Both authors read and approved the final manuscript.
